# Equilibria of Idealized Confined Astral Microtubules and Coupled Spindle Poles

**DOI:** 10.1371/journal.pone.0038921

**Published:** 2012-06-14

**Authors:** Ivan V. Maly

**Affiliations:** Department of Computational and Systems Biology, University of Pittsburgh School of Medicine, Pittsburgh, Pennsylvania, United States of America; Virginia Tech, United States of America

## Abstract

Positioning of the mitotic spindle through the interaction of astral microtubules with the cell boundary often determines whether the cell division will be symmetric or asymmetric. This process plays a crucial role in development. In this paper, a numerical model is presented that deals with the force exerted on the spindle by astral microtubules that are bent by virtue of their confinement within the cell boundary. It is found that depending on parameters, the symmetric position of the spindle can be stable or unstable. Asymmetric stable equilibria also exist, and two or more stable positions can exist simultaneously. The theory poses new types of questions for experimental research. Regarding the cases of symmetric spindle positioning, it is necessary to ask whether the microtubule parameters are controlled by the cell so that the bending mechanics favors symmetry. If they are not, then it is necessary to ask what forces external to the microtubule cytoskeleton counteract the bending effects sufficiently to actively establish symmetry. Conversely, regarding the cases with asymmetry, it is now necessary to investigate whether the cell controls the microtubule parameters so that the bending favors asymmetry apart from any forces that are external to the microtubule cytoskeleton.

## Introduction

Cells often divide symmetrically to produce two daughter cells that are of at least approximately equal size. Cell lines on which experiments are conducted in cell culture typically exhibit this kind of division. Asymmetric divisions that produce daughter cells of unequal size also abound, particularly during development and differentiation. Examples include single-cell embryos of the mussel *Unio* [1], roundworm *Caenorhabditis* [2], and leech *Helobdella* [3], *Drosophila* neuroblasts [4], and mammalian oocytes [5]. In the roundworm, for example, the first unequal division creates the larger cell that is the first somatic cell and the smaller cell that is the germline cell.

In addition to the significance of size as such, for example between the large stem cell and its small progeny [6] the division into daughter cells of unequal size may lead to an unequal distribution of specific components of the mother cell cytoplasm between the progeny. Such components may include cell fate determinants [7]. In this connection, the general notion of asymmetric cell division includes cases where the daughter cells are of equal size, yet differ in the complement of components that they inherit. For a broader review of such cases, in addition to the cited work by McCarthy and Goldstein [4], see, e.g., Knoblich [8], and Siller and Doe [9]. In this paper, the term “asymmetric division” is used exclusively in reference to the division that generates daughter cells of unequal size. This case presents an obvious challenge for biomechanical explanation.

Generally, cells divide through the middle of the mitotic spindle [10]. The spindle proper consists of microtubule bundles that connect the two spindle poles. Precise terminology is needed to formulate the theoretical question posed by the symmetric and asymmetric cell division. The line segment connecting the two poles shall be called the physical spindle axis. In the geometrical sense, this axis can be extended to define a coordinate axis that passes through the cell. The instance of the (extended) spindle axis passing through the cell center can be considered first. The paradigmatic cases, e.g. the HeLa cultured cells and the first division in *Caenorhabditis*, seem to be of this nature. The fundamental question posed by the symmetric and asymmetric cell division can then be formulated as follows. What determines coincidence of the center of the spindle proper with the cell center? What determines its shift from the cell center along the spindle axis?

Besides the microtubules of the spindle proper, additional so-called astral microtubules radiate from the two centrosomes at the spindle poles. As reviewed below (see also, e.g., Pearson and Bloom [11]), it is generally believed that the spindle is positioned through the astral microtubules’ interaction with the cell boundary. Three kinds of effects are considered: the bending elasticity of microtubules that are deformed by their contact with the cell boundary, the stochastic assembly and disassembly of the microtubules, and pulling on the microtubules by molecular motors that are anchored on the cell boundary. It must be observed that the bending elasticity is an intrinsic property–all microtubules possess it. In contrast, the stochastic assembly and molecular motors may or may not play a role, depending on the specific intracellular conditions. Therefore, the latter two mechanisms will always influence the spindle position in combination with the effects of bending elasticity. This natural hierarchy makes it imperative to understand the effects of bending elasticity for the success of integrated biomechanical understanding of symmetry and asymmetry of cell division.

The pioneering theory by Bjerknes [12] explained the salient features of spindle positioning in amphibian blastomeres. It dealt exclusively with bending elasticity, but microtubule deformations were not computed explicitly. It was assumed that straight astral microtubules radiate in all directions equally from the spindle poles. Each microtubule by assumption contacted the cell boundary with its distal end and developed the Euler buckling force that corresponded to its length. In addition to reproducing the orientations in the changing cell geometry of the progressively dividing embryo, it was demonstrated that the spindle orientation exhibited bifurcations. Alternative equilibrium orientations appeared with the elongation of the spindle relative to the cell size. The centers of the spindles, however, corresponded with the cell center. An eccentric equilibrium required that the two asters be unequal, for example, in the bending rigidity of their microtubules. The adequacy of the straight-line and buckling-force approximations and the stability of the calculated equilibria to perturbations were not assessed.

Subsequently, in a model by Théry et al. [13], the orientation of the spindle in a circular cell was treated as resulting from pulling on astral microtubules by postulated force-generating elements anchored on the cell boundary. Only situations with central symmetry were considered. Microtubule deformations and bending elasticity were not included in the model. Force-balance models of pole separation and spindle morphogenesis [14] had treated the astral microtubules similarly. The model by Grill et al. [15] that addressed the question of the asymmetric first division of *Caenorhabditis* similarly did not include microtubule bending. It was concluded that the shift of the spindle was caused by unequal distribution of the pulling elements on the cell boundary, which therefore exerted unequal force on the two poles. A different model by Grill et al. [16] dealt with the spindle-pole oscillations that are observed in the *Caenorhabditis* embryos. In this model, bending of astral microtubules against the cell boundary was included in addition to the pulling. The microtubule deformations were not explicitly computed. The force associated with it was computed using the linear Hookean dependence of the force on the distance between the pole and the boundary. The resulting oscillations of the pole in this one-dimensional model were about the middle (symmetric) position. A different model for the oscillations [17] computed the microtubule deformations explicitly in three-dimensions. The deformations were caused by the viscous drag in the cytoplasm and instantaneous pulling by the pulling elements on the ends of the microtubules that were coming in contact with the boundary. Upon contact with the boundary, the microtubules in this model disassembled, preventing development of a durable deformation of bending against the boundary.

It should be noted that in contrast with the reviewed simplifications of the treatment of the astral microtubules in spindle models, the deformations of the microtubules in the spindle proper (those that connect the two poles) have been treated with the precision of the standard bending elasticity theory [18], [19].

Thus, a general analysis of the effects of the microtubule elasticity on spindle positioning is still lacking, despite the fundamental role of this force. The present paper aims to extend and complement the reviewed approaches. The focus is on the question of symmetric vs. asymmetric positioning of the spindle, as it was posed in precise terms above. Deformations of astral microtubules are computed explicitly, and the stability of the equilibria to perturbations is assessed. The treatment proceeds from an overview of the different theoretical regimes to calculation of some sample structures that may be deemed realistic. This approach is motivated by the presently insufficient experimental data pertaining to the quantitative descriptors of mitotic spindles, and by the desire to establish the theoretical context in which the experimentally observed structures may subsequently be placed.

## Methods

In terms of its construction and numerical solution, the model is a minor extension of our previous model for the interphase [20]. Instead of one microtubule aster around the single centrosome in the interphase model, there are now two asters around the two rigidly coupled centrosomes at the spindle poles, and these asters may now be partial in the sense that microtubules may not radiate in all directions. There are no other changes from the previous model, to which the reader is referred for details that are not deemed essential to repeat here. The essential details are given below, and the new model elements described fully.

As is well known, the material spindle poles are small compared with the cell size, and the spindle proper which connects them contains a large mass of crosslinked microtubules [10]. In view of this, in the model the spindle proper is an absolutely rigid segment that connects two points in space that represent the poles (centrosomes). In this respect, the model is similar to the discussed models by Bjerknes [12], Kozlowski et al. [17], and Théry et al. [13]. The length of the spindle proper (the interpolar distance) is denoted *S*.

**Figure 1:**
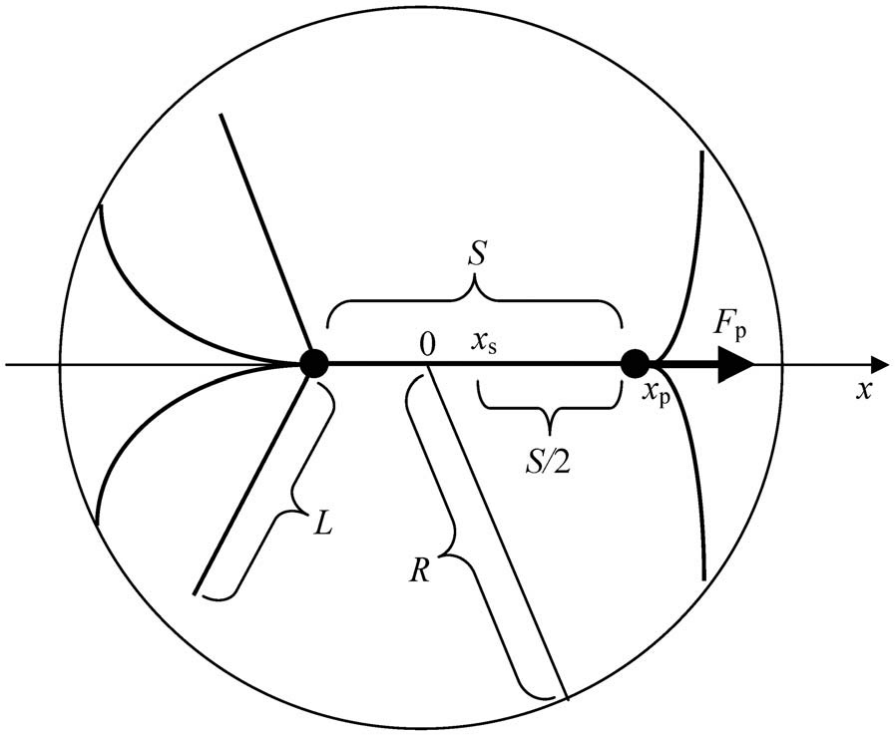
Model and notation.

To address the problem as posed in the introduction, only axially symmetric situations will be considered, in which the axis of symmetry coincides with the spindle axis (i.e. passes through the poles). In the Cartesian coordinate system of the model, the *x* axis is collinear with the spindle axis. When isolated spindle poles are considered, and unless otherwise noted, the pole on the right is meant. The pole coordinate is denoted *x*_p_; the coordinate of the middle of the spindle proper is *x*_s_. *F*_p_ is the projection on the *x* axis of the total force acting on an isolated pole. *F*_s_ is the projection on the *x* axis of the total force acting on the spindle (i.e. on both poles). Both forces are collinear with the *x* axis due to the axial symmetry. Fig. 1 illustrates the model and notation.

Like the microtubules in the earlier interphase model [20], the astral microtubules are assumed to be rigidly clamped at the centrosomes, and their contact with the spherical cell surface is frictionless. The bending of the microtubules is calculated according to the standard beam-bending equation, as described in detail in the earlier paper [20]. The lowest-energy equilibrium solution is calculated, because the alternative equilibria are unstable in three dimensions. The standard beam-bending equation is generally adequate in application to microtubules [21]. Examples of more advanced models for bending of single microtubules can be found in the work of Brangwynne et al. [22] and Gu et al. [23]. Microphotographs in the papers cited in the introduction will confirm that microtubules in mitotic cells do not converge on spindle poles at sharp angles in a fan-like arrangement. This rules out the alternative hinged boundary condition on the centrosome. The smooth bending forms argue in favor of equilibrium flexure and against additional constraints.

As in the earlier interphase model [20], microtubules are assumed to be clamped on the centrosome in equally spaced directions. To obtain the total force acting on the centrosome, the force calculated for each direction is integrated with the constant density of microtubules per unit solid angle at the centrosome. Unlike in the fully isotropic centrosome in the interphase model, the spindle model takes into account the fact that the more inward-pointing microtubules at the spindle poles become part of the spindle proper, and the more outward-pointing ones are the astral microtubules. Accordingly, the angle θ between the clamped direction and the outward direction of the spindle axis does not exceed θ_max_.

The microtubule bending rigidity is denoted *EI*, and the total number of microtubules that radiate from the pole is *N*. The microtubule length is *L*, and the cell radius is *R*. Intrinsically, the model cytoskeleton structure is centrally symmetric (*EI, L*, *N*, and θ_max_ are equal for both poles). No assumptions are made a priori about parameter values. As demonstrated below, the model behavior as it concerns the problem posed in the Introduction is controlled entirely by three compound parameters (*L/R*, *S/R*, θ_max_), in the space of which the presented analysis is, for practical purposes, exhaustive.

The task of finding the equilibrium conformation of the microtubule cytoskeleton is divided into finding the equilibrium forms of microtubules and finding the equilibrium position of the centrosome. The following equations specify the model for the single microtubule. They consist of the standard equilibrium beam equation, and of the boundary conditions of clamping on the centrosome and frictionless contact with the cell boundary. *x* and *z* are the Cartesian coordinates in the plane of the microtubule. *s* is the axial coordinate in the microtubule, *θ* is the angle of the microtubule. *β* is the angular coordinate of the microtubule end, and *P* is the contact force. Δ is the deviation of the centrosome from the center of the cell.
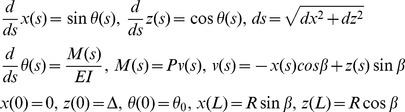



Since
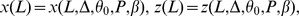



the contact conditions specify the unknown parameters *P* and *β* as the functions.
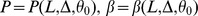



In three dimensions there is only one stable solution [20], and, correspondingly, one (*P*, *β*) pair. With the known parameters (*P*, *β*), the differential equations of bending determine the deformed shape of each microtubule and the stresses in it. In particular, the action of each microtubule on the centrosome will be known. For each microtubule, we will have the component of the force.
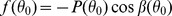



that contributes to the total force on the centrosome. Introducing the angular density of microtubules, *p*, the total force on the centrosome is found as.
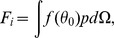



where the integration is over the solid angle of the microtubule emanation from the spindle pole *i*. The equilibrium condition for the spindle is.
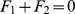



The previously derived [20] analytical approximation is used as a starting guess for the equilibrium solution corresponding to the central position of the pole. This solution is then refined numerically using the ballistic method. The same method is then used to continue the solution with respect to *Δ*. Variable step sizes are used to accurately account for the singularity associated with the microtubule contact with the cell boundary.

## Results

The model computes forces acting on the individual pole and on the spindle as a whole that arise from bending of the astral microtubules against the cell boundary, as described in detail and defined rigorously in the Methods section. The numerical results are presented graphically by plotting the force on the pole (*F*_p_) against the position of the pole (*x*_p_). Similarly, the results pertaining to the entire spindle are presented graphically by plotting the force on the spindle (*F*_s_) against the position of the middle of the spindle *x*_s_. The functions so plotted, *F*_p_(*x*_p_) and *F*_s_(*x*_s_), are referred to as the pole and spindle force functions. To present the results in their parameter-independent form, the position coordinates are normalized by presenting them as a fraction of the cell radius *R*. Similarly, the forces are normalized by dividing by the force that is characteristic of the cell with the given parameters. As established by the model (Methods), the relevant parameters are the number of astral microtubules *N* and the bending rigidity of a single microtubule *EI*. The characteristic force is then *N EI*/*R* ^2^. The plots of the force functions are at a glance revealing of the static equilibria, because in an equilibrium, the force equals zero.

### Limiting Case θ_max_ = 0

**Figure 2:**
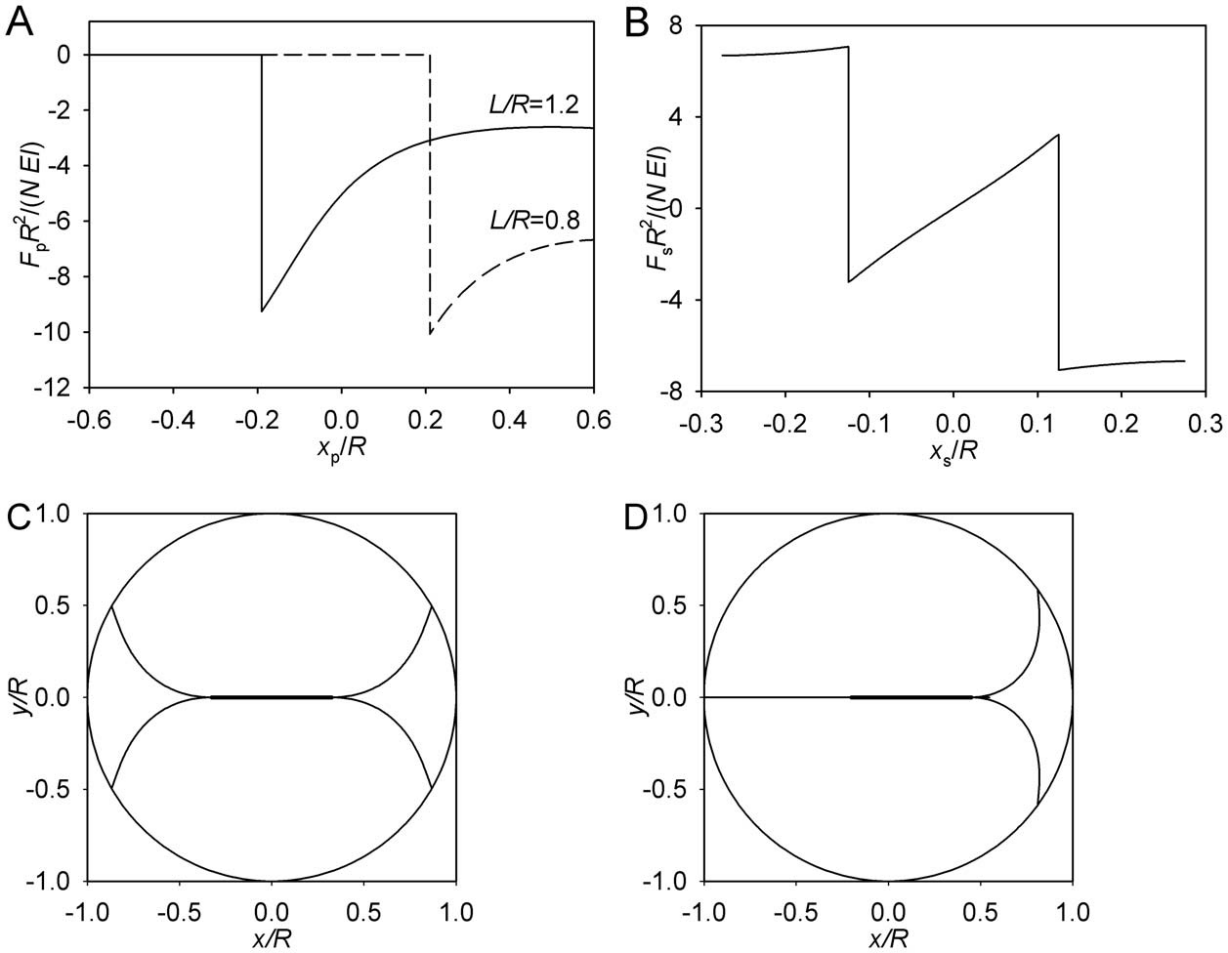
Limiting case of θ_max_ = 0. (A) Pole force function. (B) Spindle force function. (C) Symmetric equilibrium. (D) Asymmetric equilibrium. B–D: *L* = 0.8 *R*, *S* = 0.65 *R*. For clarity, only two microtubule forms are plotted. These microtubules lie in the (*x*,*y*) plane that passes through the spindle axis. The circumference is the section of the cell surface, and the thicker line segment depicts the spindle proper.

It is expedient to consider first some limiting cases that set the theoretical context for the mechanics of the more biologically relevant regimes. The simplest behavior is exhibited by the structural case of astral microtubules that emanate from the pole exclusively along the axis of the spindle (θ_max_ = 0). As the pole moves collinearly with the spindle axis, the microtubules will abut on the cell boundary, buckle, and bend, exerting force on the pole. A sample calculation is presented in Fig. 2. When the axial distance of the pole from the boundary is greater than the length of the astral microtubules, the force is zero. When the distance is equal to the length, the magnitude of the force can take any value between zero and the buckling force. For shorter distances, the force decreases, as the increasingly bent microtubules become less efficient at resisting the displacement of the pole. When the movement of two such poles is coupled through the spindle proper, three regimes are possible. If the spindle proper is short, the astral microtubules may not come in contact with the boundary, and the symmetric position will be a neutral equilibrium. This is only possible with short microtubules, whose length *L* satisfies the following relation to the spindle length *S* and cell radius *R*: *L*+*S*/2<*R*. The special case of the entire structure just fitting in the cell without deformation (*L*+*S*/2 = *R*) is unlikely to be realized. When *L*+*S*/2 is greater than *R*, microtubule emanating from one pole or from both must be bent.

Consider the symmetric case, in which both sides are bent equally. This, obviously, is an equilibrium. However, the magnitude of the force exerted on the pole is in this situation a locally decreasing function of the pole’s distance from the boundary (Fig. 2). Therefore movement of both poles to the right will decrease the magnitude of the force exerted by the boundary on the right pole. By symmetry, the magnitude of the force exerted on the left pole will be increased. The symmetric equilibrium proves unstable. The coupled poles will continue moving spontaneously, until the new equilibrium is reached. In this other equilibrium, the force of the bent microtubules acting on one pole will be balanced by the force of the straight microtubules acting on the other pole. This is always possible, because the magnitude of the force of a bent microtubule is always lower than the buckling force. The asymmetric equilibrium is stable. Indeed, movement in the direction of the bent microtubules will leave the opposite pole unsupported, as the straight microtubules lose contact with the boundary. Movement in the opposite direction will place the system in a state it already passed during its spontaneous movement from the unstable symmetric equilibrium.

Thus, in this special case, we observe instability of symmetry, stability of asymmetry, and the possibility to predict the stable conformation from the structural parameters that include the length of the spindle *S* and the length of the astral microtubules *L*, relative to the cell radius *R*. The forces are proportional to the microtubule bending rigidity *EI* and to the number of the microtubules *N*, but the positions and stability of the equilibria do not depend on these parameters. The more biologically relevant cases that are considered below exhibit more complex behavior but retain the fundamental aspects illustrated here.

### Limiting Case θ_max_ = π

**Figure 3:**
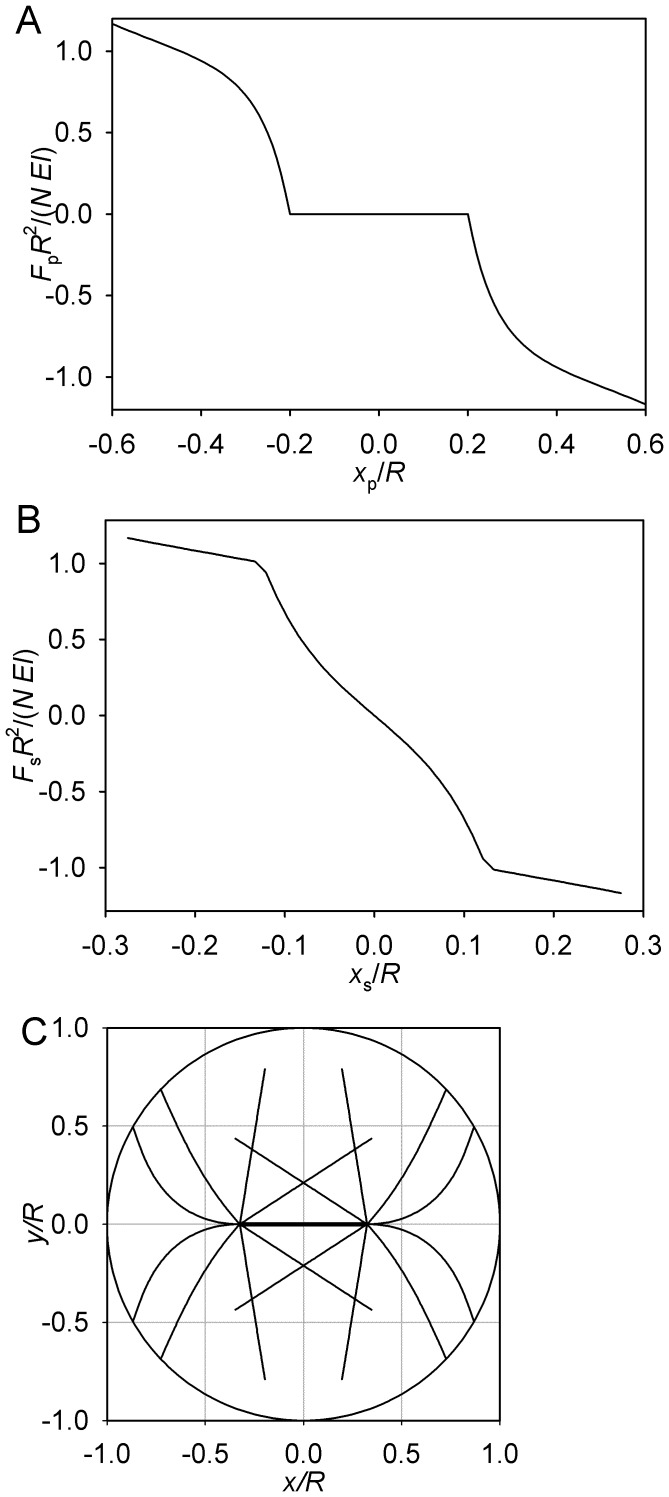
Limiting case of θ_max_ = π, short microtubules. (A) Pole force function. (B) Spindle force function. (C) Equilibrium conformation. *L* = 0.8 *R*, *S* = 0.65 *R*. For clarity, few microtubule forms are plotted. These microtubules lie in the (*x*,*y*) plane that passes through the spindle axis. Their values of θ are sampled uniformly between 0 and θ_max_.

The opposite extreme case is also revealing–the special case of complete, intrinsically spherical asters at each pole (θ_max_ = π). Now the behavior depends on whether the astral microtubules are longer or shorter than the cell radius. The case of short microtubules is simple. After the aster comes in contact with the boundary, the force exerted on the pole increases gradually with *x*_p_. The graduality is due to the number of the microtubules in contact with the boundary increasing gradually in this case, as compared with the case of θ_max_ = 0. In addition, only the axial microtubules (θ = 0), whose contribution to an aster with θ_max_≠0 is infinitesimal, go through developing the buckling force during the axial movement of the spindle; all others deflect on contact in a continuous manner. The bending leads to the decrease in stiffness, as can be seen in Fig. 3. The decrease in stiffness of the aster (the progressively shallower slope of the force curve) is, however, different from the above-considered case, where the very magnitude of the elastic force decreased with the progressing deformation. The numerical results (Fig. 3) indicate that the softening effect of the deformation (Fig. 2) in the case of the complete aster is more than offset by the increasing numbers of microtubules that come in contact with the boundary: Although each progressively bending microtubule exerts a progressively lower force, the number of the bending microtubules grows so rapidly that the total force is incresing. Even though the total force is a nonlinear function of the pole position, the force resisting the outward movement of the pole is monotonic. In this sense it is similar to simple linear (Hookean) elasticity. Thus, a spindle with two complete asters of short microtubules exhibit stability of symmetry. In addition, the monotonicity means that there is only one equilibrium conformation of the mitotic microtubule cytoskeleton, insofar as the latter is large enough to maintain contact with the cell boundary.

**Figure 4:**
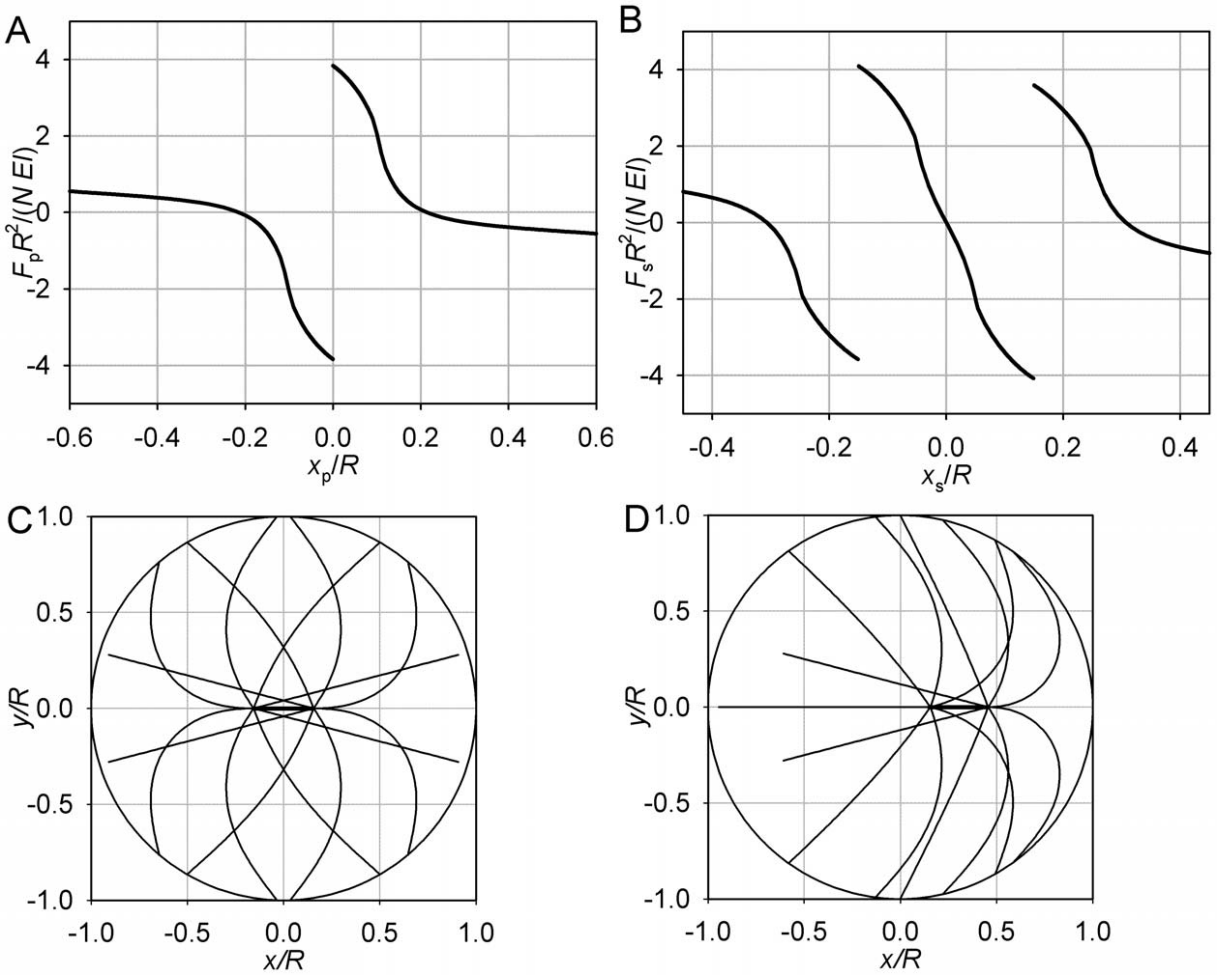
Limiting case of θ_max_ = π, long microtubules. (A) Pole force function. (B) Spindle force function. (C, D) Equilibrium conformations. *L* = 1.1 *R*, *S* = 0.3 *R*. Plotting conventions as in Fig. 3.

The positioning of an isolated complete aster with comparatively long microtubules was considered in our interphase model [20]. In this case, considered now in application to the isolated spindle pole, the pole force function has a root at approximately *x*_p_ = 2(*L*–*R*): a more centrally positioned pole is attracted to the cell margin, while a more eccentrically positioned one is repelled. Considering now the spindle with such an aster at each pole, one can observe that the presence of the root does not affect the stability of symmetry. Like in the case of short microtubules (Fig. 3), the pole force function is still monotonically decreasing for *x*_p_>0, albeit it now changes sign (Fig. 4A). Thus, the symmetry of the spindle will be stable.

Overall, however, the pole force function now is not monotonic (Fig. 4A), opening the possibility of additional equilibria if both poles are on the same side of the cell. The system remains attracted to the symmetric equilibrium until the spindle proper is moved entirely into one half of the cell. Then the conformational transition described in the earlier article [20] occurs in the more central aster and places the system on the other branch of the spindle force function (Fig. 4B). The discussed monotonicity of each half of the single pole force function ensures that the spindle now spontaneously continues in the previously forced direction. Indeed, even if the more distal pole is already in the region where it is repelled by the boundary, the more central pole upon crossing the center always experiences a greater force repelling it from the center. The new equilibrium will be reached when the two forces become equal in magnitude. This strongly asymmetric equilibrium will also be stable. This is due to the piece-wise monotonicity: whether the poles are on different sides or on the same side of the cell, the force function (Fig. 4A) is locally decreasing for each pole. A centripetal movement of the spindle from the asymmetric equilibrium increases its repulsion from the center and decreases its repulsion from the nearest boundary, and vice versa. The spindle will therefore return to the asymmetric equilibrium, unless the forced movement brings one of the poles to the opposite half of the cell, in which case it will spontaneously move to the symmetric equilibrium.

### Transitions between Limiting Cases

**Figure 5:**
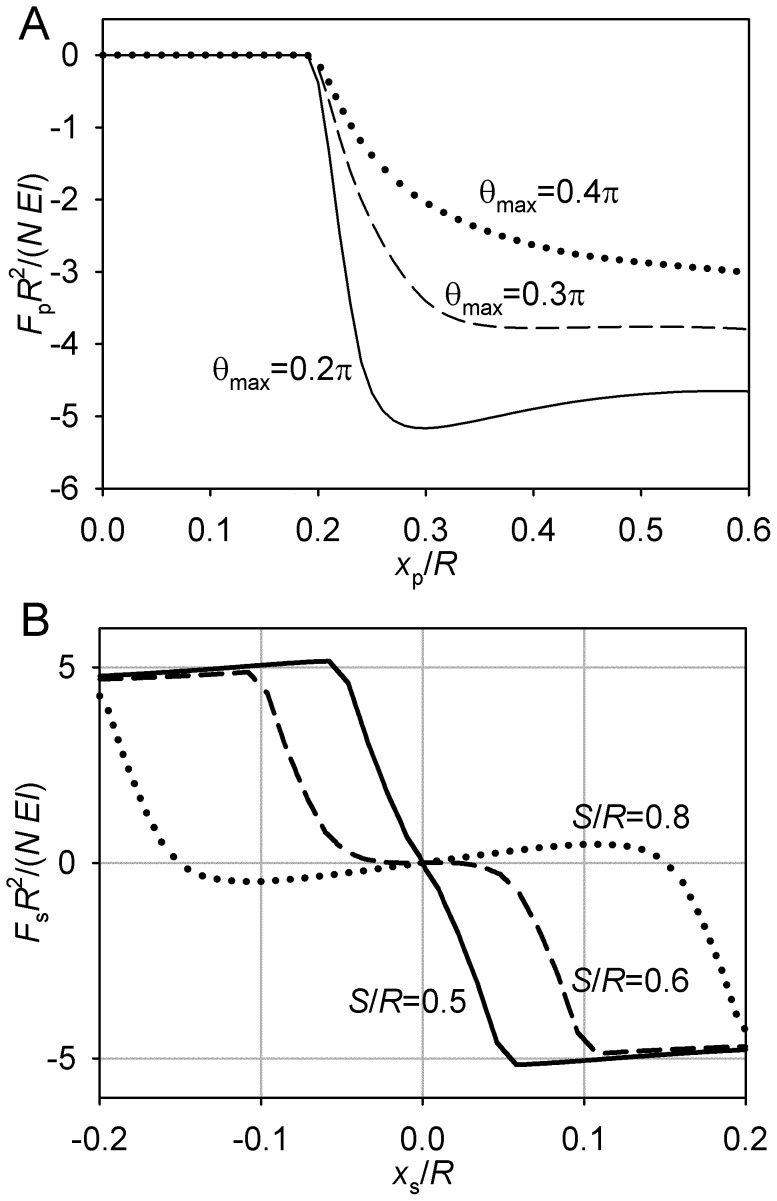
Transition between θ_max_ = 0 and θ_max_ = π in the case of short astral microtubules. *L* = 0.8*R*. (A) Pole force function. (B) Spindle force function, θ_max_ = π/5.

Let us first consider the case of short microtubules. We have seen that as θ_max_ increases from 0 to π, stability of symmetry and nonexistence of asymmetric equilibria replace instability of symmetry and stability of asymmetry. Computations demonstrate that increasing θ_max_ from zero first makes the development of the extremum pole force smooth (Fig. 5A, solid curve; cf. Fig. 2). The finite interval of *x*_p_ in which the pole force function (Fig. 5) is decreasing emerges immediately when θ_max_ exceeds zero. The range of values of *S* that place the symmetric poles in these intervals will correspond to stable symmetry. Unlike in the extreme case of θ_max_ = π, however, the pole force function is increasing beyond the extremum (Fig. 5A). Let us denote the pole position that corresponds to the extremum *x*_p_’. For *S* exceeding 2*x*_p_’, the behavior seen with θ_max_ = 0 is retained, and symmetry is unstable (Fig. 5B). The corresponding stable asymmetric equilibrium is derivative from the one described in the case of θ_max_ = 0: one pole occupies a position that lies toward the cell center from the pole force function extremum, and the other occupies a position toward the cell margin from that pole’s extremum. With further growth of θ_max_, the aster increases resistance at more eccentric pole positions (Fig. 5A, dashed curve). This eventually erases the nonmonotonicity of the pole force function. Fig. 5A shows that for sufficiently large θ_max_ the function becomes monotonic as in the case of θ_max_ = π (cf. Fig. 3). The spindle now possesses only one equilibrium, which is symmetric and stable. Thus with short astral microtubules, there is an abrupt transition at some intermediate θ_max_ to the behavior that is qualitatively like that seen with θ_max_ = π.

**Figure 6:**
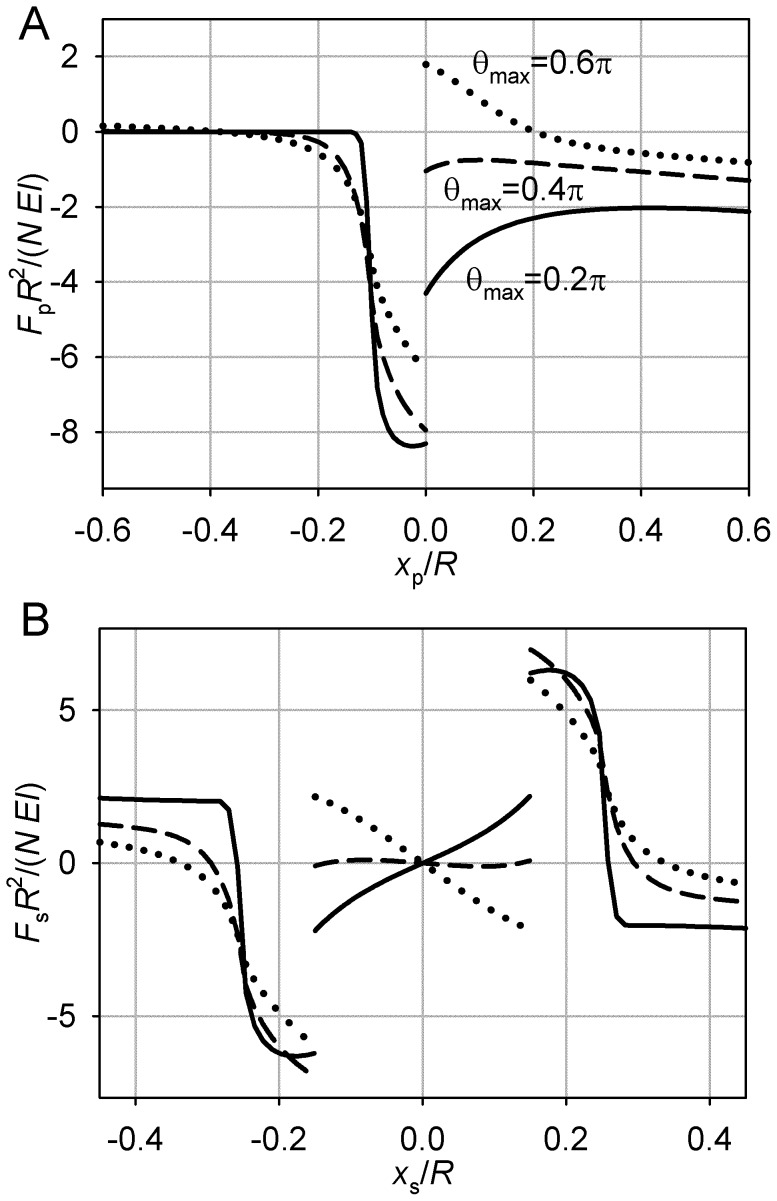
Transition between θ_max_ = 0 and θ_max_ = π in the case of long astral microtubules. *L* = 1.1 *R*. (A) Pole force function. (B) Spindle force function. *S* = 0.3 *R*. The line styles correspond to values of θ_max_ as in panel A.

With long microtubules, the increase of θ_max_ first leads to the changes observed with the short microtubules, namely the smoothing of the pole force function extremum (Fig. 6A, solid curve; cf. Fig. 2). In addition, the function in this case develops a discontinuity. It is caused by the conformational transition between the lowest-energy conformations that differ on the two sides of *x*_p_ = 0 and were described in the previous paper [20]. The transition was non-observable in the case of θ_max_ = 0 because the alternative conformations in that degenerate case were equivalent. A more consequential difference from the case of the short microtubules is that the long microtubules are more deformable, and the drop of the force magnitude past the buckling-force maximum that they exhibit is much deeper. Because of this, the increase in resistance at large pole displacements in this case cannot erase the nonmonotonicity efficiently. Instead, at around θ_max_ = π/2 the pole force function develops two descending branches (Fig. 6A). The difference from the already considered limiting case of complete asters (Fig. 4A) is that the pole force function with the intermediate θ_max_ may not change sign across the discontinuity. This reflects the comparatively simple behavior of asters with θ_max_<π/2: the individual pole would find equilibrium when all microtubules that are emanating from it are straight, unlike in the more complete asters, in which bending is unavoidable. Despite this difference in the individual behavior of separate poles, poles coupled by the spindle will behave in the same way as with the more complete asters of long microtubules, because of the fundamental similarity of the pole force function with the two descending branches. The symmetric position is now stable, as is the asymmetric equilibrium (Fig. 6B, dashed curve).

### Special Case *L* = *R*

The intermediate case of *L*≈*R* presents special interest. Theoretically, it connects the qualitatively different types of behavior already described for long and short microtubules. Biologically, astral microtubules seem to be comparable in length with the cell radius in the morphogenetically important instances of large cells of early embryos [1]–[3], [15]. Evidently, the case of *L* = *R* is by itself unrealistic, because the two quantities cannot be exactly equal. This special case, however, establishes a useful reference in the space of the model parameters and regimes of behavior.

**Figure 7:**
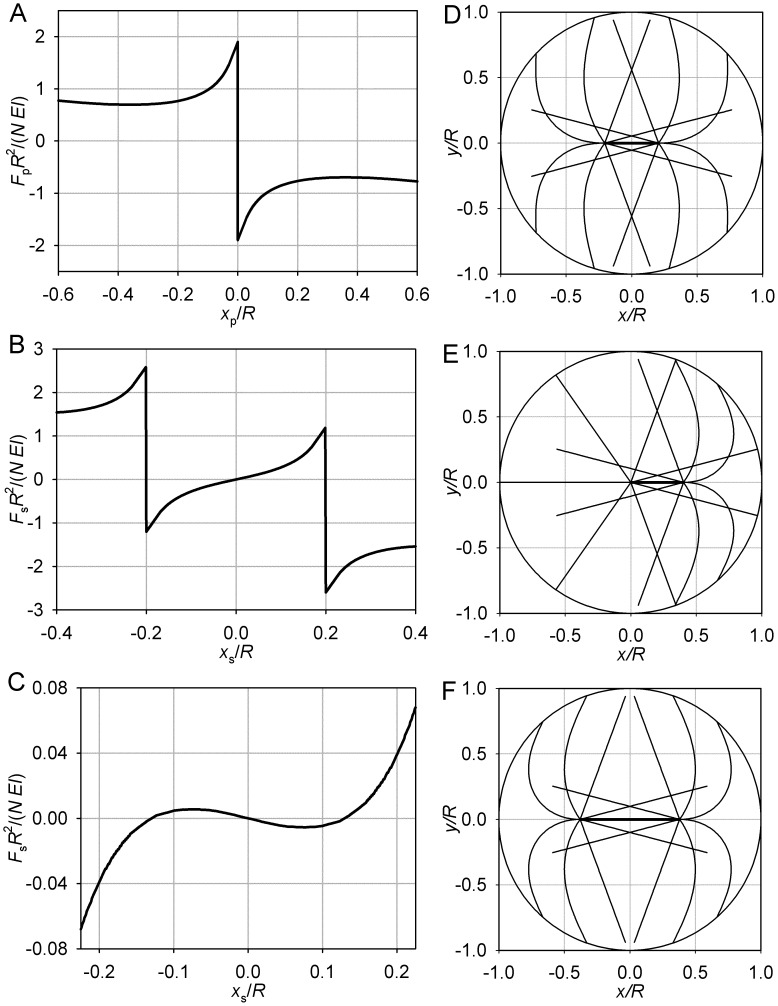
Special case of *L* = *R*, θ_max_ = π. (A) Pole force function. (B) Spindle force function, *S* = 0.4 *R*. (C) Spindle force function, *S* = 0.75 *R*. (D) Unstable symmetric equilibrium, *S* = 0.4 *R*. (E) Stable asymmetric equilibrium, *S* = 0.4 *R*. (F) Stable symmetric equilibrium, *S* = 0.75 *R*. Plotting conventions as in Fig. 3.

Calculations show that a complete (θ_max_ = π) aster with *L* = *R* develops the peak force just before its pole reaches the cell center (Fig. 7A). This force is associated with the buckling force of the microtubules that all straighten when the pole reaches the center. When the pole is in the center, the elastic force can take any value between the positive and negative extremum, and will be zero for an aster not subjected to any external force. Thus, an individual separate aster exhibits a special kind of stability of the central position, in which a finite restoring force develops upon an infinitesimal perturbation.

However, the magnitude of the restoring force decreases with the magnitude of the perturbation (Fig. 7A). A pole that is closer to the center therefore experiences a higher centripetal force than a pole farther away from the center. This is a condition for instability of symmetry of the spindle. One pole’s centripetal movement will be completed at the expense of the increasingly eccentric position of the coupled pole. This is demonstrated by the spindle force function (Fig. 7B). In the asymmetric equilibrium, which is stable, the force on the eccentric pole is balanced by the equal force supported by unbuckled microtubules of the centrally positioned pole.

The described behavior is observed for spindles with interpolar distances not exceeding a certain value. Inspection of the pole force function (Fig. 7A) shows that the stiffening of the aster at large eccentricities of the pole creates a range of extreme pole positions where the magnitude of the force is increasing with the distance from the center. This creates conditions for stability of symmetry of sufficiently long spindles (Fig. 7C).

### Overview of Parameter-space Domains

**Figure 8:**
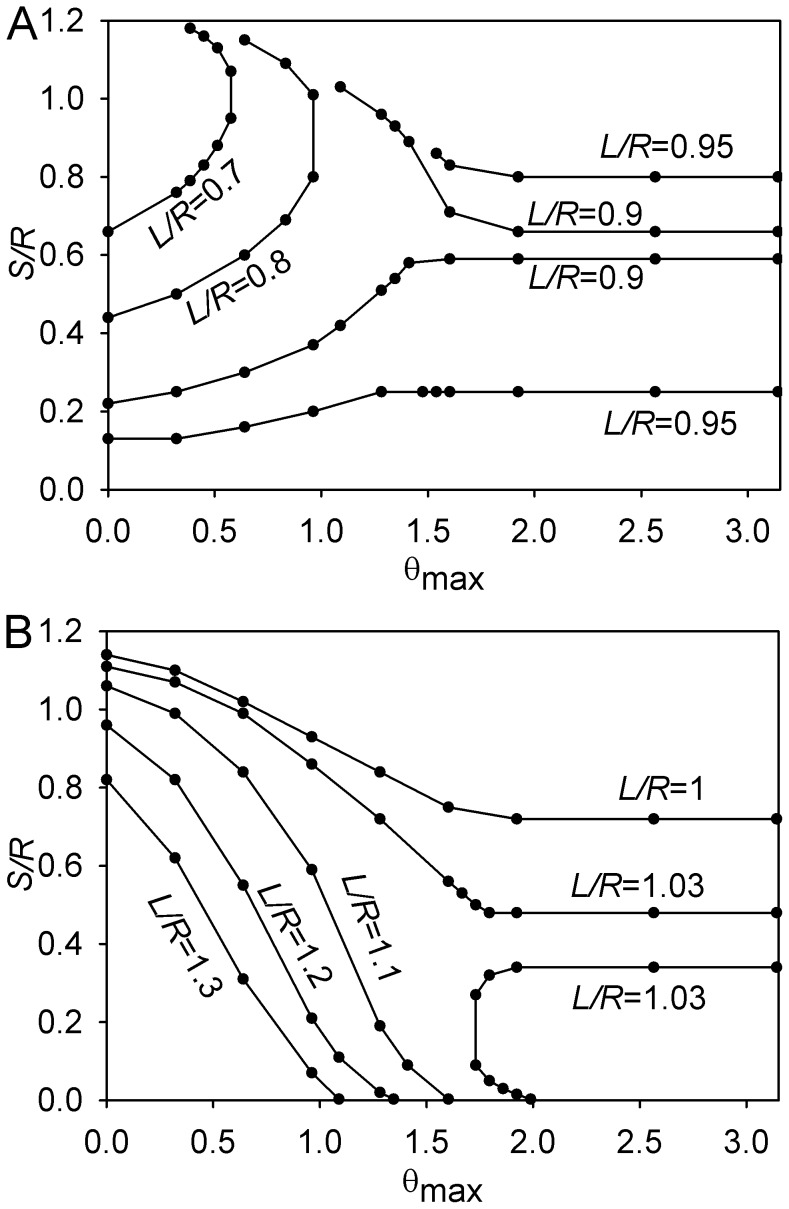
Symmetry instability domain. In the three-dimensional space of structural parameters (θ_max_, *S/R*, *L/R*), *L/R* is understood as the vertical dimension in this figure. Isolines corresponding to the indicated values of *L/R* are shown. (A) Surface bounding the domain from below. (B) Surface bounding the domain from above. (These contour plots may be viewed as topographic maps. In A, symmetry is stable when the point representing the conditions of the individual cell lies below the mapped surface, and unstable otherwise. In B, symmetry is unstable when the point lies below the mapped surface, and stable otherwise.).

The domain of instability of the spindle symmetry in the three-dimensional space of the structural parameters (θ_max_, *S/R*, *L/R*) is outlined in Fig. 8. The behavior described in the preceding sections can be seen in its outline alongside some additional features that have not been described in the text. The boundaries of the domain show how the continuity between the regimes characteristic of the short and long microtubules is achieved through the special case of *L* = *R*. Specifically, for both long and short microtubules, the (θ_max_, *S/R*) cross-sections of the symmetry instability domain are restricted to θ_max_<π/2.

For short microtubules, longer spindles exhibit unstable symmetry. Note, however, that the stability of spindles that are particularly short (*S*<2(*R*–*L*)) is only neutral in this case, because in their symmetric position, the astral microtubules do not contact the boundary. For long microtubules, on the contrary, shorter spindles exhibit instability of symmetry. In this case, it is of note that the longer spindles exhibit bistability between the symmetric and asymmetric equilibria, as was illustrated in Fig. 6B (dashed curve).

For *L*≈*R*, the range of instability of symmetry extends through θ_max_ = π. This behavior, which has been illustrated in the special case of *L* = *R*, is retained for *L* taking values between approximately 0.9 and 1.05 *R*. Although this range is narrow in absolute terms, it seems to be particularly relevant, because inspection of images in the literature suggests that *L* is often similar to *R* in the morphogenetically important instances of large cells in early embryos (1–3,15).

### Interpretation of Parameter-space Domains: Length Distributions

It can be seen that if each microtubule length from a set supports stability of symmetry, any distribution of lengths over that set will also support stability of symmetry. Conversely, if each length by itself supports instability, any distribution will also support instability. Additionally, if any subset supports neutral stability, the outcome is dictated by the complementary subset. With this in mind, inspection of the parameter-space domains makes predictions about the stability of symmetry that will be obtained with microtubule length distributions.

As described in the preceding section, in the (θ_max_, *S/R*, *L/R*) space the instability domain is bounded and embedded in the domain of stability (Fig. 8). Furthermore, it lies above the domain of neutral stability (*S/R* <2*L/R*). At large θ_max_, there is a considerable separation between the domains of instability and neutral stability; at small θ_max_, they almost touch.

The support of a microtubule length distribution is represented in the said parameter space by a vertical segment. If such a segment lies entirely within or outside the instability domain, the central symmetry will correspondingly be unstable or stable. For example, any distributions whose support falls entirely to the right of the *L/R* = 1 isoline in Fig. 8B will predict stable central positioning of the spindle.

Further, unless the distribution is unusually sharply concentrated at the intermediate values, the instability domain can be considered as touching the neutral stability domain for smaller θ_max_. Therefore, distributions whose support falls below the upper bounding surface of the instability domain (Fig. 8B) predict instability of symmetry, if θ_max_ is not large.

In the likely case of descending exponential distributions [24], the contribution of the few exceptionally long microtubules may prove negligible. In that case, the prediction can be further simplified: For larger θ_max_ and *S/R*, stability is predicted, and for smaller θ_max_ and *S/R*, instability is predicted.

### Sample Equilibrium Structures

**Figure 9:**
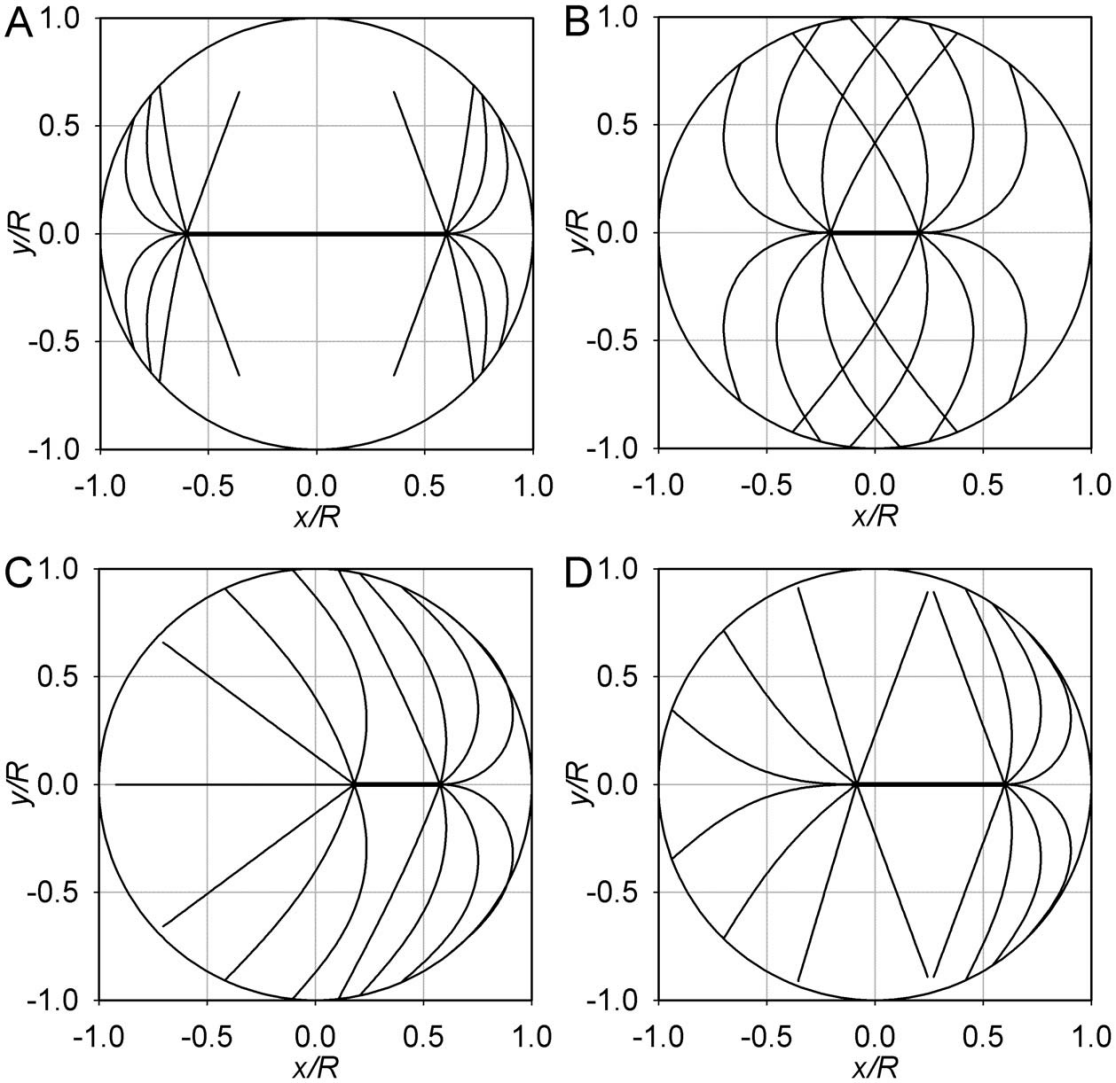
Sample equilibrium conformations. For clarity, only few microtubule forms are plotted. These microtubules lie in the (*x*,*y*) plane that passes through the spindle axis. Their values of θ are sampled uniformly between 0 and θ_max_. The circumference is the section of the cell surface, and the thicker line segment depicts the spindle proper. In all examples, θ_max_ = 0.6π. (A) *L* = 0.7 *R*, *S* = 1.2 *R*. (B) and (C) represent the alternative conformations that exist with *L* = 1.1 *R* and *S* = 0.4 *R*. (D) *L* = 0.95 *R*, *S* = 0.7 *R*.

Among the theoretically possible structures and equilibria, several can be considered paradigmatic, based on qualitative examination of images of spindles in the experimental literature. Firstly, there is the case of a long spindle with short astral microtubules that radiate from the poles in a wide solid angle. The equilibrium conformation is plotted in Fig. 9A. According to the preceding analysis, in this regime (large *S*/*R*, small *L*/*R*, large θ_max_), the symmetric equilibrium is the only equilibrium, and it is stable. Awaiting measurements motivated by this theory, it appears that this regime is common among the variety of equally dividing cells. The HeLa cultured cells are one example [13].

The second instance demonstrating applicability of the theory is the structure with long astral microtubules that radiate in a wide angle. This regime exhibits the bistability between the symmetric and asymmetric equilibria. The alternative conformations are illustrated in Fig. 9 B and C. This example is characterized by a comparatively small *S/R* and large *L/R*. In this respect it is reminiscent (in the asymmetric conformation) of the first division in the invertebrate models of development that include the mussel *Unio* and the roundworm *Caenorhabditis* (1–3,15). For comparison, Fig. 9D displays the only stable equilibrium that exists in the regime with *L* ≈ *R* and a spindle of medium length, which is asymmetric. In terms of the spindle proportions and position, it is reminiscent of the mouse oocyte (where the positioning involves F-actin [5]).

The example of bistability in particular raises the question of the absolute magnitude of the collective spindle forces. The natural unit of force, *N EI*/*R* ^2^, equals 22 pN when *N* = 100, *E* = 26 pN µm^2^, and *R* = 10 µm. The barrier for switching from the symmetric to asymmetric state in the case plotted in Fig. 6B (dotted curve) is then 65 pN. Note that the barrier for switching from the asymmetric state is higher, 156 pN. The above estimates were order-of-magnitude for *N* and *R* in a generic cell, and a mid-range experimental value [25] for EI. Equally relevant may be *N* = 1000, and *R* = 5 µm, in which case the two barriers will be 2.6 and 6.2 nN. Targeted measurements are needed to determine if such barriers (∼0.1–1 nN) can be overcome in a given cell. Accepting the experimental estimate of the force that can be developed, for example, by a single dynein motor as 2.6 pN [26], the barriers calculated here could be overcome by 25–2000 dynein molecules, which is not an unrealistic number.

## Discussion

The presented theory derives the static equilibria of the spindle from the considerations of bending of the astral microtubules against the cell boundary, and assesses the stability of the equilibria. In this respect, it is an extension of the pioneering model by Bjerknes [12]. The distinctive method of the new theory is explicit computation of the bent microtubule forms. The chief new prediction is that an intrinsically symmetric mitotic microtubule cytoskeleton may spontaneously adopt asymmetric conformations, when constrained within the cell. In this respect, the model is conceptually derivative from our earlier treatment of the interphase microtubule cytoskeleton [20] and from the pioneering work of Holy [27] on the interphase microtubule asters. The different subject of the present work is the mechanics of two coupled asters of microtubules that are found at the two poles of the mitotic spindle. The individual confined asters in the interphase models always break the symmetry with respect to the cell center. The mitotic model, at least under certain conditions, exhibits stable equilibria that are centrally symmetric, as well as bistability between the symmetric and asymmetric equilibria.

The novel theoretical possibility of the spontaneous development of asymmetry through bending of astral microtubules, and the uncovered special requirements of stability of symmetric conformations, pose new types of questions that can be asked when designing and interpreting experiments. In particular, the new theory urges not to consider symmetric spindle positioning a “default” state of the system. Just as importantly, it urges not to seek the source of the asymmetry necessarily outside the basic structure of the mitotic microtubule cytoskeleton.

When studying a case of symmetric positioning, it may be worth investigating what makes it symmetric. Do the parameters such as the length of the astral microtubules (*L*) and of the spindle proper (*S*) have values that support the stable symmetry? If they do not, what forces external to the microtubule cytoskeleton act against the collective bending forces and actively establish the symmetry?

When studying the case of asymmetric spindle positioning, it may be worth investigating the possible contribution of the collective bending effects of the astral microtubules to the generation of asymmetry. In fact, in the light of the theory this question may acquire priority. Although the quantitative nature of the collective bending that breaks symmetry is complex, the hypothesis that the source of asymmetry resides in the basic cytoskeleton structure itself is simple, compared with hypotheses that involve asymmetric regulation or asymmetric external forces applied to the structure.

The spontaneous development of asymmetry through unequal bending does not by itself have a preferred direction: Each asymmetric equilibrium in the model has a counterpart, which is mirror-symmetric about the cell center. External forces and regulation mechanisms may be responsible for biasing the spontaneous symmetry-breaking, even if they are not responsible in the specific instance for the generation of the asymmetry. Similarly, the action of the external forces, even transient, may be responsible for the selection between the symmetric and asymmetric equilibrium in the cases of multistability.

In view of the stochastic assembly of microtubules, exact symmetry is impossible. Yet it is the symmetry of the microtubule growth from the two poles that is often so striking under the microscope, whether or not the bending is symmetrical. This underscores the necessity to assess the stability of the symmetric conformations, because apart from any external forces, the small intrinsic asymmetries will always take the system out of the exact symmetric equilibrium.

The presented model deals with the collective mechanical behavior of a large number of long microtubules, which are capable of bending strongly without pivoting on their attachment points at the spindle poles. These conditions are not met in yeast, where there is only a small number of short microtubules or bundles, which may pivot [28]. Attempting to isolate the effects of the microtubule bending, the model purposefully disregards the deformability of the cell cortex within which the microtubules are confined. The complete system of the deformable cortex and the interphase microtubule cytoskeleton was analyzed in our earlier numerical model [29], [30]. It similarly exhibited the emergence of asymmetry. Future models should address such difficult problems as the astral microtubule bending with force-dependent microtubule assembly [31], [32] and with microtubule-based motors [11], whose action on the microtubule cytoskeleton depends on the microtubule conformation [33]. Spindle positioning likely depends on the quantitative effects of the interaction between these forces.

The model deals with equilibria only, not with dynamics. Although the elastic force can be calculated using this model for every intermediate conformation, the velocity of movement under the action of these forces will depend on the resistance to the movement. The cited work [18], [33] computed the dynamics of movement of explicitly modeled, deformed microtubule cytoskeletons driven by molecular motors, assuming viscous resistance with certain effective viscosity coefficients. The likely order-of-magnitude similarity of the collective elastic forces and ones produced by molecular motors (see above) argues that depending on the actual resistance, either both should fail to produce a reasonable displacement speed or both should succeed. The microtubule lifetime in mitotic cells is ∼1 min [34], [35], indicating that the collective bending force is not shared between a fixed set of microtubules. Rather, this force should be sustained by the steady-state structure consisting of rapidly renewed individual microtubules.

Summarizing, the explicit numerical treatment of bending of astral microtubules indicated existence of new types of collective mechanical behavior in mitotic cytoskeletons, which include symmetry-breaking and multistability. The goal of this publication is to identify new types of questions that can be asked in experimental work and establish a quantitative framework that can guide experiment design. Interpretation of new experimental results, and, possibly, re-interpretation of those previously obtained, will require generalization of the numerical model and its rigorous adaptation to the structural features of each specific cell type.
